# Visitors’ Motives for Attending a Healthy Food Exhibition

**DOI:** 10.3390/ijerph17082703

**Published:** 2020-04-15

**Authors:** Yahua Bi, Sooyoung Choi, Insin Kim

**Affiliations:** Department of Tourism and Convention, Pusan National University, Pusan 46241, Korea; yahuabi@pusan.ac.kr (Y.B.); sychoi@pusan.ac.kr (S.C.)

**Keywords:** visitor’s motives, satisfaction with the healthy food exhibition, memory

## Abstract

Environmental issues (i.e., food safety and environmental pollution) have increased concerns about individuals’ health as well as global environment. These concerns drive awareness for the influences of healthy foods, and eventually lead them to visit healthy food exhibitions. This research aims to understand the attendees’ motives for participating in a healthy food exhibition. Specific objectives are to identify crucial visitors’ motives influencing satisfaction with the healthy food exhibition and to verify whether visitors’ satisfaction with the exhibition enhances their memory for the experience in the exhibition. The survey was conducted by targeting visitors who participated in the Busan International Food Expo, and the data collected from 363 attendees were analyzed using the SPSS and AMOS statistical programs. The analysis results revealed that three dimensions of healthy food exhibition motives, namely perceived healthiness, perceived hedonism and perceived food safety, increase visitors’ satisfaction with the healthy food exhibition, and that satisfaction with the healthy food exhibition further had a positive impact on the visitors’ memory for the exhibition experience.

## 1. Introduction

The recent global trends related to physical and mental wellness and an increasing personal health-related consciousness partly caused by personal stress and environmental pollution have raised awareness about the importance of healthy eating in daily life and thus changed consumers’ food purchase preferences [1,2,3]. Accordingly, healthy foods have become increasingly desired as consumers’ food selection criteria have shifted from taste and price to health-related food information such as nutrition and food materials and ingredients [4]. In line with this trend, consumers’ desires for more information about healthy food products, services, and/or manufactures drive them to attend food exhibitions [5].

Exhibitions are public events at which participating organizations or companies promote themselves [6,7] by introducing their products or services and providing attendees with opportunities to experience them for marketing purposes. By attending exhibitions, consumers are able to quickly learn and obtain information about and experience a variety of new products or services [8]. In other words, an exhibition is not just a pavilion, but also an interactive place in which exhibitors communicate with attendees, and attendees experience connections with exhibitors and their tangible and intangible products and acquire solutions to their problems that help them with future purchasing decisions.

As a place for an efficient and cost-effective communication channel for business to attract consumers [9], healthy food exhibitions provide participating companies with the opportunity to showcase their healthy food products such as low-fat, low-calorie, health-benefiting, and organic foods, in an attempt to introduce and promote their products to exhibition visitors. The increasing interests in healthy eating enhance consumers’ purchase intentions for healthy foods [10,11] and tend to motivate them to attend healthy food exhibitions.

Maintaining existing visitors and attracting new ones are useful strategies for exhibition organizers to succeed in an increasingly competitive marketplace [5,9]. As exhibitions have become an important source of information for consumers, and exhibition visitors tend to attend an exhibition with different motives [12], understanding visitors’ motives for attending the exhibition and gratifying their needs are crucial for exhibition organizers and managers to plan exhibition programs [13,14]. Therefore, when exhibitors and organizers understand exhibition visitors’ motives for an exhibition, they are better placed to develop “a more effective customer relationship management” [12] (p. 404). By considering exhibition visitors’ motives, the exhibitors and organizers can utilize resources more powerfully to improve performance when preparing for the exhibition [15].

Additionally, researchers argue that understanding the motives for attending an event can help monitor satisfaction in the exhibition industry [13]. As a vital evaluation factor of the quality of a service or product [16], consumer satisfaction is a key element that businesses need to achieve for their long-time survival and success in the market [17,18]. In the Meetings, Incentives, Conventions, and Exhibitions (MICE) industry, it is also found to be a powerful antecedent for visitor loyalty [19]. Most exhibitions are held in fixed locations [13] and their success mostly relies on the number of revisit attendees and the attendees’ word of mouth communication and recommendation behaviors, which are heavily influenced by satisfaction with exhibition experiences [20]. Therefore, it is emphasized that the future fate of an exhibition depends on visitor satisfaction with exhibition experiences [21].

Researchers report that it is necessary to explain the memory phenomenon in the exhibition industry [22,23]. Memory can greatly influence customer judgments and decisions [23,24,25]. As such, prior research highlights the positive relationship between memory and behavioral intentions [23,26,27,28]. For instance, memory established on past experience with a certain tourist destination cognitively affects a tourist’s present destination selection [29] and thus encourages tourists to return to the destination [28].

This study proposes a conceptual model to understand visitors’ motives for attending a healthy food exhibition. Specifically, this study attempted (1) to identify key visitors’ motives influencing visitor satisfaction and (2) to verify whether visitor satisfaction with a healthy food exhibition induces a positive memory related to visitors’ experiences with the healthy food exhibition.

Limited research has examined visitors’ motives in a healthy food exhibition setting. Previous studies on visitors’ motives for attending exhibitions have mainly focused on individual factors (e.g., escape and novelty) and exhibition factors (e.g., service quality) [9,15,30]. Therefore, it would be practical and meaningful to investigate what motives drive visitors to participate in a healthy food exhibition. Additionally, given the importance of memory in determining consumer choices and behaviors, the results of this study should provide valuable implications for food exhibition organizers and participating companies to develop marketing strategies to attract exhibition visitors and retain revisit attendees and suggestions for future research.

## 2. Literature Review

### 2.1. Visitors’ Motives

Visitors’ motives refer to attendees’ desires to see the expected products or services at the exhibitions [9,31]. Understanding the motives of participating in the event helps to establish an effective product development strategy [5]. By attending exhibitions, participants can quickly learn, compare and ultimately obtain new products or services [8]. Environmental issues (i.e., food safety and environmental pollution) have increased concerns about individuals’ health [1,2,3] as well as global environment, making a number of corporates try to enhance their green image by making efforts for decreasing environmental damages [32,33,34,35,36]. These concerns drive awareness for the influences of healthy foods, and eventually lead them to visit healthy food exhibitions. Motives for exhibitions have been investigated in various studies, such as trade exhibitions [37], handicraft exhibitions [7], and consumer travel exhibitions [38].

Researchers argued that people will have different motives when choosing healthy foods [3,10,39,40], so it is appropriate to consider the motives for healthy food exhibitions as a multidimensional variable. Teng and Lu [3] divided healthy food motives into three classes: health consciousness, food safety concern, and ecological motives. Hwang [39] explained the motives for consuming healthy food and suggested four dimensions: self-presentation, food safety concern, environmental concern, and having an ethical consumer identity. In a study on organic food identity and behavior, Hansen, Sørensen, and Eriksen [40] investigated three motives: environmental consciousness, health consciousness, and social consciousness. Bauer, Heinrich, and Schäfer [10] categorized four dimensions of healthy food purchase motives and found that organic labels could influence consumers’ perceptions of their major purchase motives for global, local, and private brands. Table 1 illustrates existing studies on motives for healthy food consumption. Developed from the previous research, the present study explores visitors’ motives as a multidimensional construct containing four types: perceived healthiness, perceived hedonism, perceived environmental friendliness, and perceived food safety.

Perceived healthiness can be defined as the perception that visiting an exhibition can improve one’s health and lifestyle [10,41]. Consumption of health-related products reflects people’s concerns about the living environment, their own health, and animal welfare [42]. Eating healthy food is also an important factor in determining human health [43]. As such, researchers highlighted that perceived healthiness affects consumer evaluation and choice of products [41], which are all important indicators of market development and promotion of healthy eating [44].

Perceived hedonism means that visitors can enjoy a pleasant experience and improve their well-being when visiting a healthy food exhibition [10]. Didier and Lucie [45] stated that people might buy health-related organic foods because this process can lead to hedonism. While people are pursuing healthy food, they also pay attention to the pleasure brought by food; therefore, health should be connected with hedonism [46]. Consequently, hedonism from healthy foods may drive people to participate in related exhibitions.

Perceived environmental friendliness relates to the many environmental problems associated with food consumption, such as climate change, exhausted resources, and unhealthy eating habits [47]. Environmental friendliness has become an important issue for discussion in the exhibition industry in order to achieve sustainable development [48]. Environmental friendliness provides an opportunity for people to improve environmental conditions [10]. Understanding environmentally friendly products and related environmental protection resources has become an important motive for people to visit the exhibition.

Perceived food safety refers to the visitors’ perception that the exhibition’s products are free of chemical residues, pesticides, pollution, and are safe to eat [3,10]. Food safety is closely linked to corporate reputation and marketing [49]. Additionally, the perception of food safety positively affects people’s purchase behavior of healthy food [50,51]. Perceived food safety can positively predict health food involvement [3], which in turn can promote people’s intention of purchasing healthy food.

### 2.2. Satisfaction with a Healthy Food Exhibition

According to the expectation–confirmation theory [52,53], satisfaction is an important predictor generating post-exposure affect and purchase intention [54,55,56,57,58]. When the perceived performance of a product or service meets customer expectations, the confirmation that appears leads to satisfaction [59] and such satisfied customers will have the intention to repurchase and vice versa [52].

In a similar vein, satisfaction with the food exhibition means that visitors are satisfied with the visit to the exhibition and believe that the visit meets their expectations. Researchers found a strong link between satisfaction and loyalty among MICE participants [19,20,59]. Severt, Wang, Chen, and Breiter [20] demonstrated that attendee satisfaction is a predictor for return intention and word-of-mouth in the context of the regional conference. Kim and Malek [19] revealed that visitors’ satisfaction could positively impact convention loyalty. The above research indicated that satisfaction is a key factor for the promotion of market development and business survival [17].

Among the factors affecting satisfaction, motive explains the original intention of the visitor to choose the exhibition, while satisfaction involves gratification with a good emotional reaction to meet the intention [21,60]. Additionally, the motives of visitors are closely linked to the content of the exhibition. Severt, Wang, Chen, and Breiter [20] found that the motivation for educational benefits positively affects the satisfaction of visitors participating in the conference. In the context of the exhibition, Lee [61] verified that information resources and educational benefits greatly affect visitors’ satisfaction.

The findings denote that motives for attending exhibitions have a positive relationship with visitors’ satisfaction [20,61]. Although previous research has investigated motives as a factor influencing satisfaction, it is meaningful to explore the motives of a healthy food exhibition generating visitors’ satisfaction with the exhibition since the relationship between the motives for a healthy food exhibition and satisfaction with a healthy food exhibition has been rarely investigated. According to the attitude–behavior theory [62], consumer behavior or behavioral intention is positively influenced by consumer attitude. As mentioned before, different motives for healthy food consumption are proved to positively relate to consumer intention to purchase healthy food [3,10,41,42,45,50,51] presumably increasing consumer satisfaction. Likewise, the gratification of motives for attending the exhibition would elicit exhibition visitors’ positive attitudes towards the exhibition and leads to favorable behaviors. Hence, the current study posits that motives are positively related to satisfaction with the healthy food exhibition as follows:

**Hypothesis** **1 (H1).** *Perceived healthiness increases satisfaction with the healthy food exhibition.*

**Hypothesis** **2 (H2).** *Perceived hedonism increases satisfaction with the healthy food exhibition.*

**Hypothesis** **3 (H3).** *Perceived environmental friendliness increases satisfaction with the healthy food exhibition.*

**Hypothesis** **4 (H4).** *Perceived food safety increases satisfaction with the healthy food exhibition.*

### 2.3. Memory

In the context of the healthy food exhibition, memory is when participants remember positive things from the exhibition and leave with good and unforgettable experiences. Visitors can relive memorable experiences by utilizing mindfulness [63]. Memory gained from past experiences can have an impact on product judgments [25], consumer decisions [24], loyalty [27], and behavioral intentions [26,64]. What is more, creating a memorable experience for visitors at the event plays an indispensable role in market development [65], such as determining the revenue-generating capabilities of a company [66].

In the tourism industry, Kim and Jang [67] emphasized that management should provide excitement (e.g., scents and music) so that visitors can form memories of cultural events. Cahill and McGaugh [68] noted that emotional arousal could affect memory. The high emotional reaction encourages individuals to remember details of relevant events and retain vivid memories [64,69,70]. Satisfaction involves the gratification of a positive emotional reaction [60,71]. Hence, satisfaction can serve as an efficient way to affect memory. Ali, Ryu, and Hussain [26] validate this relationship by demonstrating that memory of creative tourists’ experience has a positive impact on their satisfaction with the destination. Based on the above discussion, this research posits that satisfaction with the healthy food exhibition positively affects visitors’ memories.

**Hypothesis** **5 (H5).** *Satisfaction with the food exhibition increases memory.*

**Figure 1:**
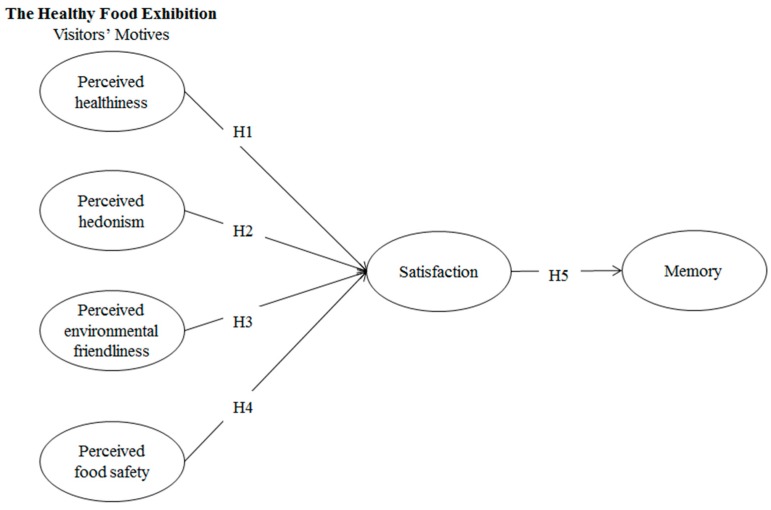
The research model.

According to the aforementioned discussion, the current study presents the research model shown in Figure 1. 

## 3. Method

### 3.1. Measures

The study instruments were derived from previous studies pertaining to visitor motives, satisfaction with healthy food exhibitions, and memory. In particular, visitors’ motives were explored in the four dimensions described above with sixteen items that were developed from Bauer, Heinrich, and Schäfer [10]. Three items to measure satisfaction were slightly modified for the exhibition industry from scales employed by Back and Parks [72] and Kim and Ok [73]. Memory was operationalized with three items from Oh et al.’s [74] study based on visitors’ self-evaluation of participation experience in the exhibition. All items were rated on a 5-point Likert scale ranging from 1 strongly disagree to 5 strongly agree.

To ensure content validity, three experts who are conducting research and working in the exhibition field were invited to evaluate the appropriateness of the measures. To confirm the face validity, a pilot test was performed on graduate students, frequent visitors to the exhibition, and organizers of the exhibition center. The suggestions from experts and professionals were considered in revising and designing the questionnaire.

### 3.2. Data Collection and Sample

**Figure 2:**
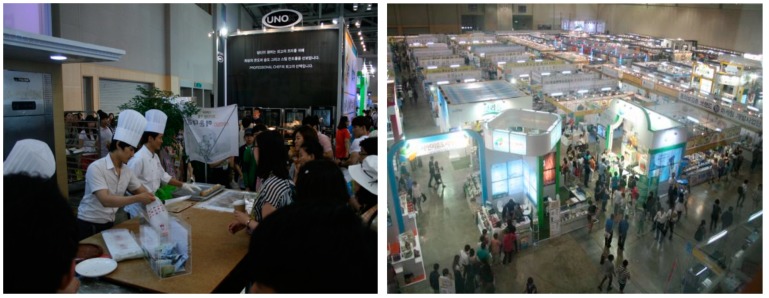
The pictures of the 22nd Busan International Food Expo.

The Busan International Food Expo is a festival that promotes and popularizes Korean healthy food to people globally. The exhibition aims to develop new tourism resources through food and cultural activities and to understand people’s needs for food. With the increasing concern for healthy living [75,76], the Busan International Food Expo has won the support of enterprises and visitors and has become the largest food exhibition in South Korea outside Seoul [77]. The 22nd Busan International Food Expo, held at BEXCO for four days, attracted 338 companies from 15 countries [78], and 51,246 attendees were participated in various taste-testing events, food-making processes, experience activities, and healthy food knowledge (see the Figure 2).

This study conducted an onsite survey for four days during the Busan International Food Expo. Four graduate students majoring in tourism and convention in South Korea joined in the data collection after training. The target population was the individuals who attended the food exhibition. To collect the data, surveyors asked visitors leaving the exhibition center after attending food expo in the exit of BEXCO to fill out a self-administered questionnaire using the convenience sampling method. To express gratitude, the respondents who successfully completed the questionnaire received a small gift (bottled water/cookies). Finally, a total of 363 valid questionnaires were used for data analysis. Table 2 details the profile of the respondents.

### 3.3. Data Analysis Tools

The research model was analyzed using statistical package programs of SPSS version 23.0 (IBM, New York, NY, USA) and Amos version 23.0. (IBM, New York, NY, USA). Before analyzing the data, we conducted descriptive statistic to assess normality of our data. As presented in Table A1, descriptive statistics revealed that values of skewness and kurtosis are in the range of threshold (from −2.58 to +2.58), indicating acceptable normal distribution of the data [79].

Structural equation modeling (SEM) was used based on two stages suggested by Anderson and Gerbing [80]. First, the measurement model was analyzed to confirm whether constructs and items used in this research are valid and reliable by conducting confirmatory factor analysis (CFA). Second, the structural model was analyzed to clarify causal relationships between the constructs in this study.

## 4. Results

### 4.1. Measurement Model Validation

The validity and reliability tests were undertaken through CFA. The CFA analysis results showed that the measurement model indicated acceptable model fit indices (χ^2^ = 408.216, df = 194, χ^2^/df = 2.104 at *p* < 0.001, goodness-of-fit index (GFI) = 0.909, comparative fit index (CFI) = 0.965, incremental fit index (IFI) = 0.965, Tucker–Lewis index (TLI) = 0.958, and root mean square error of approximation (RMSEA) = 0.055 [81]). Convergent validity for the sets of items was assessed by confirming the values of factor loadings for items and average variance extracted (AVE) for constructs based on the recommendation of Fornell and Larcker [82]. Table 3 shows that all standardized factor loadings indicated a higher value than the cut-off of 0.5 and were significant at *p* < 0.001. The AVE values for all constructs exceeded the threshold of 0.5 [79]. Therefore, convergent validity was successfully confirmed. 

According to Fornell and Larcker [82], discriminant validity can be estimated by comparing the minimum AVE value of pairwise with the squared correlation. Each construct confirmed to be distinctive, except for two pairs: perceived healthiness–perceived hedonism, and perceived hedonism–satisfaction. Therefore, the chi-square difference test was conducted for these two pairwise based on Bagozzi and Yi [83]. A combined model was compared to an uncombined model, and the analysis results revealed that each construct was different from one another. Composite reliability for all latent constructs surpassed the threshold value of 0.7, ranging from 0.882 to 0.938 as indicated in Table 4. Thus, all constructs showed internal consistency.

### 4.2. Structural Model Testing

Structural modeling was conducted to estimate the relationships among latent constructs. The model fit indices showed acceptable levels (χ^2^ = 426.168, df = 198, χ^2^/df =2.152 at *p* < 0.001, GFI = 0.906, CFI = 0.963, IFI = 0.963, TLI = 0.956, and RMSEA = 0.056) [79].

As presented in Table 5, perceived healthiness (β = 0.160, *p* < 0.05), perceived hedonism (β = 0.561, *p* < 0.01), and perceived food safety (β = 0.140, *p* < 0.05) exerted significantly positive effects on satisfaction, supporting H1, H2, and H4. However, the impact of perceived environmental friendliness on satisfaction was not significant, and hence H3 was not supported. Visitors’ satisfaction with the healthy food exhibition had a positive effect on memory (β = 0.796, *p* <0.01), and hence hypothesis 5 was supported.

## 5. Discussion and Implications

This study proposed a research model (1) to identify determining visitor motives for attending healthy food exhibitions influencing visitor satisfaction with the healthy food exhibition, and (2) to investigate whether satisfaction as perceived by visitors of healthy food exhibition lead positive memory for exhibition experiences. The SEM results provided valuable evidences that the proposed motives for attending healthy food exhibitions (barring perceived environmental friendliness) affect visitor satisfaction, thereby contributing to form positive memory. Accordingly, the findings of this study contribute to motive research for a successful healthy food exhibition from the visitors’ perspectives by examining the relationships between visitors’ motives, satisfaction, and memory for the exhibition. The findings have several implications.First, by adopting the multidimensional structure of consumer motives from a prior study [10], this study derived the critical visitor motives for attending a healthy food exhibition and extends the exhibition and motive literature. Specifically, this study empirically tested four dimensions of exhibition visitors’ motives in a healthy food exhibition setting and confirmed the reliability and validation of the construct. The results also provide empirical evidence demonstrating that the four-dimensional construct of exhibition visitors’ motives is effective in understanding visitors’ attitudes toward exhibition experiences and further behavioral intentions. Therefore, organizers and exhibitors of a healthy food exhibition can utilize the measures of motives that were used in this study in order to examine whether the exhibition they hold truly meets visitors’ expectations and/or needs.

Second, the study results support the findings of previous studies [20,61,84] that visitors’ motives positively influence visitors’ satisfaction with a healthy food exhibition. Specifically, the findings revealed that perceived healthiness motive, perceived hedonism motive, and perceived food safety motive have positive impacts on visitors’ satisfaction. Among the three significant motives, perceived hedonism had the strongest effect on satisfaction, while the other two motives showed a positive but weak effect on satisfaction. On the other hand, the results failed to find a significant relationship between perceived environmental friendliness motive and satisfaction. Such results indicate that knowing what exhibition attributes or service elements visitors expect from a healthy food exhibition and gratifying the visitors’ expectations will generate visitors’ positive evaluations of the exhibition experiences. Therefore, participating companies or organizations of a healthy food exhibition may improve exhibition performances through the following suggestions in order to increase visitors’ satisfaction. When referring to perceived healthiness, exhibitors or organizers of healthy food exhibitions need to promote the health-related benefits of the healthy food products introduced in the exhibition. The positive relation of perceived hedonism motive to satisfaction shows that visitors of a healthy food exhibition not only seek food-related information but also pursue positive and affective responses by attending the exhibition. Striving to provide attendees with a feeling of pleasure and a sense of gratification from attending a healthy food exhibition is of importance in generating visitors’ satisfaction with the exhibition experiences. Additionally, as people become more concerned about food safety issues, exhibitors can inform visitors that their healthy food products are pollution-, chemical-, and pesticide-free, and feature high food safety.

Third, the findings of this study provide new insights into the role of memory in the context of healthy food exhibitions. To date, there has been a limited understanding of memory as a predictor of visitors’ attitudes toward consumer experience and behavioral intentions. Although scholars have pointed out that a better understanding of memory helps to effectively design and organize exhibitions [85], the concept of memory in the healthy food exhibition sector has remained unclear. Participants are eager to remember their meaningful moments and retain evidence related to those meaningful moments [86]. The results of this study suggest that exhibitors can enhance visitors’ positive memories with exhibition experiences by increasing the visitor perceptions of healthiness, hedonism, and food safety related to healthy foods products or services and thus heighten visitors’ satisfaction with the exhibition experiences. Such interaction between exhibitors and visitors helps visitors form deep impressions related to exhibition experiences, resulting in positive and unforgettable memories.

The research has certain limitations. First, although the visitors’ interest and preferences for healthy food choices might vary depending on their demographic characteristics, such as age, gender, and occupation, the current research did not differentiate between such cohorts. Therefore, the future study needs to examine differences of satisfaction influenced by motivation across demographic subgroups. Second, since the concept of memory might be treated as an important predictor of behavioral intentions [27,64], future research needs to investigate its effects on behavioral intentions such as exhibition loyalty and product purchase intentions in the exhibition.

## 6. Conclusions

Investigating the motives to participate in healthy food exhibitions is conducive to successfully holding exhibitions in the future and can provide references for companies participating in exhibitions. For exhibition organizers, it is essential to identify attendees’ motives that enhance satisfaction with the healthy food exhibition, and how attendees improve their memories about the healthy food exhibition experience. Due to social pressure, living conditions, environmental pollution, food safety, etc., people are paying increasing attention to the intake of healthy food [4], which makes them participate in healthy food exhibitions [5] as an attempt to maintain their health. In this study, a conceptual model was developed to empirically test how exhibition visitors’ motives influence their satisfaction and, thus, the formation of their positive memory with exhibition experiences in a healthy food exhibition setting. SEM assessed the research hypotheses using data collected through the onsite survey conducted in a healthy food exhibition and revealed significant positive relationships between motives (healthiness, hedonism, and food safety) and satisfaction, and satisfaction and memory. The research findings highlighted the awareness of healthy food exhibition visitors’ motives, as well as the importance of gratification of the visitors’ motives to induce satisfying and memorable exhibition experiences. Consequently, the current study enhanced the understanding of event attendees’ perceptions and attitudes in a healthy food exhibition setting and offers researchers and exhibition organizations/participating companies valuable insights into products and services features showcased in a healthy food exhibition that suit exhibition visitors’ desires.

## Figures and Tables

**Table 1:** Summary of constructs of the motives for healthy food consumption in previous research.

Study	Research Context	Proposed Construct	Outcome Variable
Bauer, Heinrich, and Schäfer [10]	Organic food brand purchase decision, compared to non-organic food brand, among German food consumers	Perceived healthiness Perceived hedonism Perceived environmental friendliness Perceived food safety	Organic food purchase intention Willingness to pay a price premium
Hansen, Sørensen, and Eriksen [40]	Organic food consumption motivations influenced by personal values among Danish food consumers	Health consciousness Environmental consciousness Social consciousness	Organic food identity Intentional organic food behavior
Hwang [39]	Relative importance of organic food buying motives among U.S. older food consumers	Self-presentation Food safety concern Environmental concern Ethical consumer identity	Organic food purchase intention
Teng and Lu [3]	Organic food consumption motives in Taiwanese food consumers	Health consciousness Food safety concern Ecological motives	Involvement in organic food Purchase intention

**Table 2:** Profile of respondents.

Characteristics	Categories	Frequency (N)	Percentage (%)
Gender	Male	226	62.3
	Female	137	37.7
Age	Under 20	28	7.7
	20s	167	46.0
	30s	60	16.5
	40s	32	8.8
	50s	45	12.4
	Older than 60	31	8.6
Level of Education	Some high school	11	3.0
	High school graduate	67	18.5
	College graduate	85	23.4
	University graduate	170	46.8
	Post-graduate	30	8.3
Occupation	Students	126	34.7
	Housewives	40	11.0
	Office workers	90	24.8
	Self-employed	31	8.5
	Professionals	27	7.5
	Others	49	13.5

**Table 3:** Confirmatory factor analysis (CFA): items and loadings.

Items	Loading	AVE
Perceived healthiness		0.716
The consumption of products in this exhibition enhances my health.	0.790	
I believe that this exhibition enables me to live healthily.	0.884	
I am of the view that the consumption of products in this exhibition has a health-promoting effect.	0.861	
This exhibition and a health-conscious lifestyle match well.	0.847	
Perceived hedonism		0.572
By participating in this exhibition, I can pamper myself.	0.733	
I can indulge myself by participating in this exhibition.	0.759	
Participating in this exhibition can affect my well-being positively.	0.817	
It is a pleasure to participating in this exhibition.	0.714	
Perceived environmental friendliness		0.692
The production of this exhibition goes easy on resources.	0.793	
I am of the opinion that during this exhibition the environment is highly valued.	0.827	
The products in this exhibition are environmentally friendly.	0.856	
This exhibition and environmentalism match well.	0.852	
Perceived food safety		0.634
I feel that the food presented in this exhibition is free of chemical residues.	0.753	
I am of the opinion that the food presented in this exhibition is not contaminated.	0.821	
The food ingredients presented in this exhibition are pesticide-free.	0.829	
I believe that the food presented in this exhibition features high food safety.	0.782	
Satisfaction		0.675
Overall, I am satisfied with this exhibition.	0.857	
I am happy with attending this exhibition.	0.865	
I believe I did the right thing when I visited this exhibition.	0.865	
Memory		0.657
I will have wonderful memories of this exhibition.	0.930	
I will remember many positive things about this exhibition.	0.917	
I will not forget my experience at this exhibition.	0.841	

Notes: All factor loadings were significant at *p* < 0.001; AVE = average variance extracted.

**Table 4:** Descriptive statistics and associated measures.

Construct		Mean	SD	CR	1	2	3	4	5	6
**1**	Perceived healthiness	3.386	0.717	0.938	1.00					
**2**	Perceived hedonism	3.409	0.693	0.882	0.811 (0.657)	1.00				
**3**	Perceived environmental friendliness	3.241	0.715	0.931	0.663 (0.439)	0.731 (0.534)	1.00			
**4**	Perceived food safety	3.458	0.683	0.914	0.598 (0.357)	0.612 (0.374)	0.740 (0.547)	1.00		
**5**	Satisfaction	3.520	0.755	0.897	0.722 (0.521)	0.806 (0.649)	0.665 (0.442)	0.610 (0.372)	1.00	
**6**	Memory	3.521	0.836	0.904	0.658 (0.432)	0.714 (0.509)	0.618 (0.381)	0.558 (0.311)	0.771 (0.594)	1.00

Note: SD = standard deviation; CR = composite reliability; Squared correlations are in parentheses.

**Table 5:** Standardized coefficients.

Paths			Standardized Estimate	*t*-Value	Support
Perceived healthiness	→	Satisfaction	0.160	2.027 *	Support
Perceived hedonism	→	Satisfaction	0.561	5.701 **	Support
Perceived environmental friendliness	→	Satisfaction	0.065	0.848	Not support
Perceived food safety	→	Satisfaction	0.140	2.175 *	Support
Satisfaction	→	Memory	0.796	16.552 **	Support

Note: * *p* < 0.05, ** *p* < 0.01.

## Appendix A

**Table A1:** Normal distribution tests.

Constructs	Items	Mean	SD	Skewness	Kurtosis
Perceived healthiness	The consumption of products in this exhibition enhances my health.	3.342	0.827	−0.001	−0.192
	I believe that this exhibition enables me to live healthily.	3.405	0.800	0.053	−0.128
	I am of the view that the consumption of products in this exhibition has a health-promoting effect.	3.416	0.780	0.036	−0.039
	This exhibition and a health-conscious lifestyle match well.	3.383	0.834	−0.156	0.561
Perceived hedonism	By participating in this exhibition, I can pamper myself.	3.240	0.831	0.252	−0.051
	I can indulge myself by participating in this exhibition.	3.477	0.832	−0.070	−0.278
	Participating in this exhibition can affect my well-being positively.	3.383	0.844	−0.016	−0.113
	It is a pleasure to participating in this exhibition.	3.537	0.864	−0.115	−0.271
Perceived environmental friendliness	The production of this exhibition goes easy on resources.	3.231	0.791	0.136	0.200
	I am of the opinion that during this exhibition the environment is highly valued.	3.218	0.824	0.143	−0.032
	The products in this exhibition are environmentally friendly.	3.339	0.816	0.069	0.221
	This exhibition and environmentalism match well.	3.176	0.832	0.180	0.187
Perceived food safety	I feel that the food presented in this exhibition is free of chemical residues.	3.457	0.870	−0.045	−0.449
	I am of the opinion that the food presented in this exhibition is not contaminated.	3.410	0.772	−0.132	−0.083
	The food ingredients presented in this exhibition are pesticide-free.	3.507	0.784	0.081	−0.412
	I believe that the food presented in this exhibition features high food safety.	3.460	0.787	−0.056	−0.081
Satisfaction	Overall, I am satisfied with this exhibition.	3.526	0.798	−0.217	0.244
	I am happy with attending this exhibition.	3.410	0.863	0.033	−0.298
	I believe I did the right thing when I visited this exhibition.	3.625	0.826	−0.302	0.032
Memory	I will have wonderful memories of this exhibition.	3.504	0.935	−0.165	−0.338
	I will remember many positive things about this exhibition.	3.590	0.876	−0.263	−0.035
	I will not forget my experience at this exhibition.	3.471	0.883	−0.129	−0.065

Note: SD = standard deviation.
